# Stromal hedgehog signaling maintains smooth muscle and hampers micro-invasive prostate cancer

**DOI:** 10.1242/dmm.027417

**Published:** 2017-01-01

**Authors:** Zhaohui Yang, Yu-Ching Peng, Anuradha Gopalan, Dong Gao, Yu Chen, Alexandra L. Joyner

**Affiliations:** 1 Biochemistry, Cell and Molecular Biology Program, Weill Cornell Graduate School of Medical Sciences, New York, NY 10065, USA; 2 Developmental Biology Program, Sloan Kettering Institute, New York, NY 10065, USA; 3 Department of Pathology, Memorial Sloan Kettering Cancer Center, New York, NY 10065, USA; 4 Human Oncology and Pathogenesis Program, Memorial Sloan Kettering Cancer Center, New York, NY 10065, USA; 5 Department of Medicine, Memorial Sloan Kettering Cancer Center, New York, NY 10065, USA

**Keywords:** HH, PCa, Reactive stroma, Fibroblasts

## Abstract

It is widely appreciated that reactive stroma or carcinoma-associated fibroblasts can influence epithelial tumor progression. In prostate cancer (PCa), the second most common male malignancy worldwide, the amount of reactive stroma is variable and has predictive value for tumor recurrence. By analyzing human PCa protein and RNA expression databases, we found smooth muscle cells (SMCs) are decreased in advanced tumors, whereas fibroblasts are maintained. In three mouse models of PCa, *PB-MYC*, *ERG/PTEN* and *TRAMP*, we found the composition of the stroma is distinct. SMCs are greatly depleted in advanced *PB-MYC* tumors and locally reduced in *ERG/PTEN* prostates, whereas in *TRAMP* tumors the SMC layers are increased. In addition, interductal fibroblast-like cells expand in *PB-MYC* and *ERG/PTEN* tumors, whereas in *TRAMP* PCa they expand little and stromal cells invade into intraductal adenomas. Fate mapping of SMCs showed that in *PB-MYC* tumors the cells are depleted, whereas they expand in *TRAMP* tumors and interestingly contribute to the stromal cells in intraductal adenomas. Hedgehog (HH) ligands secreted by epithelial cells are known to regulate prostate mesenchyme expansion differentially during development and regeneration. Any possible role of HH signaling in stromal cells during PCa progression is poorly understood. We found that HH signaling is high in SMCs and fibroblasts near tumor cells in all models, and epithelial *Shh* expression is decreased whereas *Ihh* and *Dhh* are increased. In human primary PCa, expression of *IHH* is the highest of the three *HH* genes, and elevated HH signaling correlates with high stromal gene expression. Moreover, increasing HH signaling in the stroma of *PB-MYC* PCa resulted in more intact SMC layers and decreased tumor progression (micro-invasive carcinoma). Thus, we propose HH signaling restrains tumor progression by maintaining the smooth muscle and preventing invasion by tumor cells. Our studies highlight the importance of understanding how HH signaling and stromal composition impact on PCa to optimize drug treatments.

## Abstract (precis)

**Summary:** New insights are provided into the role of hedgehog signaling in maintaining prostate smooth muscle and place hedgehog as a candidate pathway of therapeutic value for treating individuals with prostate cancer.

## INTRODUCTION

Prostate cancer (PCa) is the second leading cause of cancer-related mortality in men in the United States (Siegel et al., 2016) and the second most common malignancy in men worldwide (Torre et al., 2015). Although prostate carcinoma arises from the epithelium, numerous studies have revealed the potential influence of reciprocal interactions between prostate stromal cells (fibroblasts and smooth muscle cells or SMCs) and cancer epithelial cells on tumor progression (Barron and Rowley, 2012; Franco and Hayward, 2012). For example, human prostate carcinoma-associated fibroblasts, but not normal prostate fibroblasts, induce substantial growth and neoplasia of nonmalignant human prostate epithelial cell lines in tissue recombinants in mice (Olumi et al., 1999). Furthermore, the proportion of reactive stroma within human PCa samples has prognostic value for PCa-specific death (Ayala et al., 2003, 2011). Unlike normal prostate stroma that is primarily composed of mature SMCs, the reactive stroma of human PCa has been described as enriched with myofibroblasts and fibroblasts, and depleted of mature SMCs (Tuxhorn et al., 2002). In the normal adult mouse prostate, our recent study identified four stromal subtypes: SMCs that express smooth muscle actin [SMA; also known as actin, alpha 2, smooth muscle, aorta (ACTA2)], fibroblasts scattered between prostate ducts, and two additional vimentin-expressing ductal fibroblast-like cell types – ‘wrapping cells’ that wrap the outside of the smooth muscle (SM) layer and ‘subepithelial cells’ situated between the SM and the epithelium (Peng et al., 2013). Furthermore, genetic inducible fate mapping (GIFM) studies during regeneration of the adult prostate raised the possibility that each stromal subtype has a distinct stem or progenitor cell (Peng et al., 2013). The relationship between the different stromal lineages and cancer reactive stromal cells are not known, nor whether a particular subtype is tumor protective.

The hedgehog (HH) signaling pathway plays a pivotal role in development and regeneration of the adult prostate, and abnormal HH signaling has been implicated in multiple carcinomas including PCa (Gonnissen et al., 2013; Lim et al., 2014; Peng and Joyner, 2015; Shaw and Bushman, 2007). In mammals, three HH ligands, sonic (SHH), indian (IHH), and desert (DHH), exert their function by binding to the receptor patched (PTCH), which relieves inhibition of the transmembrane protein smoothened (SMO). SMO activation leads to the formation of GLI2 and GLI3 transcriptional activators, which induce target genes including *Gli1* and *Ptch1*. Because *Gli1* expression is dependent on GLI2 and GLI3 activators, it is a sensitive readout of high-level HH signaling (Bai et al., 2002, 2004). The HH signaling pathway has stage-specific roles during prostate development (Berman et al., 2004; Peng and Joyner, 2015; Yu and Bushman, 2013). During embryonic development HH signaling acts on the mesenchyme to promote ductal extension and branching, whereas at the early postnatal stage HH plays an inhibitory role on ductal morphogenesis. In the adult mouse prostate, our previous study showed that SHH is secreted by basal epithelial cells and signals to progenitors of all four stromal subtypes (Peng et al., 2013). A separate study using an *Ihh^CreER^* knock-in allele revealed that during adult prostate regeneration *Ihh* is preferentially expressed by epithelial cells between growing buds, and functional studies indicate that IHH negatively regulates epithelial bud formation by downregulating stromal *Hgf* (Lim et al., 2014). However, it has not been addressed experimentally whether any specific function of HH signaling is involved in the stromal changes seen during PCa progression.

Several studies have provided evidence for paracrine HH signaling in human and mouse PCa (Fan et al., 2004; Ibuki et al., 2013; Shaw et al., 2009), a cellular relationship resembling the epithelial-to-stromal HH signaling in developing and adult mouse prostates (Berman et al., 2004; Peng et al., 2013). Autocrine HH signaling in PCa epithelial cells has also been reported (Chen et al., 2009; Karhadkar et al., 2004; Sanchez et al., 2004), particularly in advanced and metastatic PCa specimens (Chen et al., 2009; Sheng et al., 2004; Tzelepi et al., 2011). Given the questionable reliability of antibodies to HH pathway components, the highly heterogeneous nature of PCa, and the difficulty of effectively separating tumor cells from the stroma, we have taken advantage of mouse genetic tools to study HH signaling *in vivo* during PCa progression in mouse models.

Several recent functional studies using mouse genetic carcinoma models found that stromal HH signaling reduces pancreas and bladder cancer progression (Lee et al., 2014; Mathew et al., 2014; Rhim et al., 2014; Shin et al., 2014), consistent with the poor outcomes of HH inhibitors in pancreas cancer clinical trials (Rosow et al., 2012). Specifically, genetic deletion of *Shh* in pancreatic cancer cells decreases survival and enhances tumor progression (Lee et al., 2014; Rhim et al., 2014), and deletion of *Smo* in bladder stromal cells promotes carcinogenesis (Shin et al., 2014). In addition, pharmacological modulation of the HH pathway in mice revealed accelerated or delayed pancreatic cancer development following SMO inhibitor or HH agonist treatment, respectively (Lee et al., 2014; Rhim et al., 2014). In a xenograft model, ablation of the HH co-receptors *Gas1* and *Boc* in mouse embryonic fibroblasts (MEFs) promoted the co-injected human pancreatic cancer cell lines to grow pancreatic tumors, whereas elimination of HH signaling by deletion of *Gas1*, *Boc* and *Cdon* in MEFs inhibited pancreatic tumor growth, indicating a dose-dependent role of HH signaling in differentially regulating pancreatic cancer progression (Mathew et al., 2014). In PCa, however, functional studies using mouse models have not clarified the role of HH signaling in tumorigenesis. Whereas conditional expression of oncogenic SmoM2 in the mouse prostate epithelium does not lead to mouse prostatic intraepithelial neoplasia (mPIN) or cancer (Mao et al., 2006), xenograft experiments using PCa cell lines have indicated a pro-tumor effect of HH signaling (Fan et al., 2004; Karhadkar et al., 2004), and one study using retroviral expression of SHH in the prostate reported cancer formation (Chen et al., 2006). Given the contradictory findings for the function of HH signaling in PCa, it is important to test whether excess HH signaling in the stroma changes tumor progression in a mouse model of PCa.

We have characterized the phenotype of the stromal cells in three mouse models of PCa – a probasin*-*driven *MYC* (*PB*-*MYC*) model, a conditional *ERG* and *PTEN* genetic model (*ERG/PTEN*) and a transgenic adenocarcinoma mouse prostate (*TRAMP*) model – and found that the proportions of cells with a SM- or fibroblast-like character were distinct in each of the three models. In *PB*-*MYC* and to a lesser extent *ERG/PTEN*, but not *TRAMP* tumors, SMCs are greatly depleted, recapitulating a loss of mature SMCs seen in human PCa. Using genetic fate mapping, we found that SMCs are largely lost without contributing to the fibroblast-like reactive stroma that is increased between ducts in *PB-MYC* tumors, but contribute to the expanded SM and also give rise to a specific subset of intraductal stromal cells in *TRAMP* tumors. We found that HH signaling is increased in stromal cells in all three models, especially those adjacent to tumor cells. In *PB-MYC* and *TRAMP* tumors, *Ihh* and *Dhh* rather than *Shh* are the main ligands expressed by tumor cells. In human PCa, *IHH* is the highest expressed of the three HH genes and the level of HH signaling positively correlates with the amount of stromal gene expression. To test whether stromal HH signaling can alter PCa progression, an activated form of the HH receptor was expressed in *Gli1*-expressing stromal cells of *PB-MYC* tumors. We found that excess HH signaling in prostate stromal cells has an inhibitory effect on cancer progression, potentially owing to maintenance of SM that might prevent micro-invasion of tumors. Our studies provide new insights into the heterogeneity of stromal cells in three mouse models and in human PCa, and into the possible importance of particular stromal cell types in tumor progression, and also identify the HH signaling pathway as a candidate of possible therapeutic value for treating PCa patients.

## RESULTS

### Advanced human prostate tumors show a decrease in smooth muscle

**Fig. 1.**
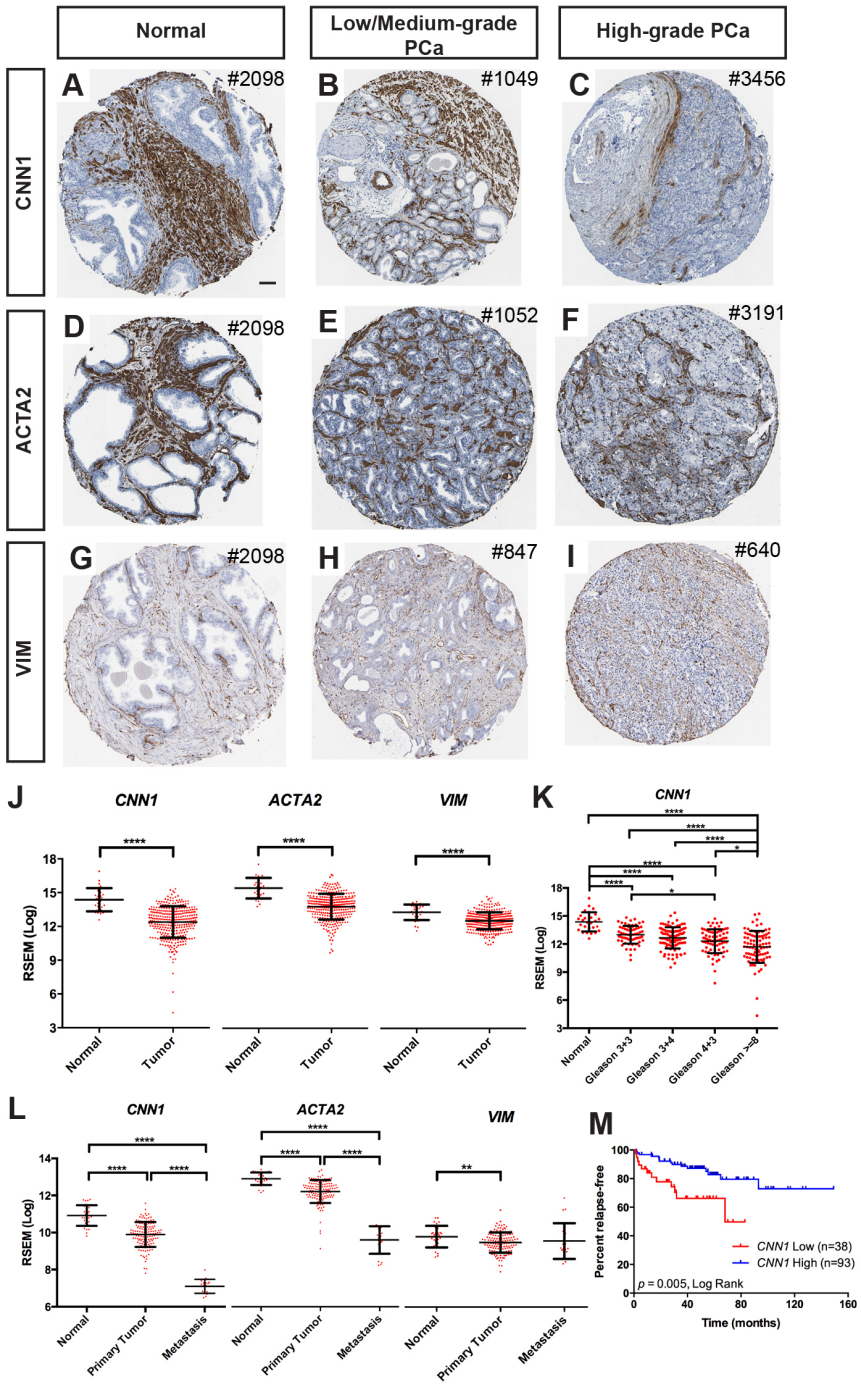
**Human prostate tumors show disruption of stromal architecture with a decrease in smooth muscle.** (A-I) Immunohistochemistry (IHC) of CNN1 (A-C), ACTA2 (D-F), and VIM (G-I) in human normal prostate, low- or medium-grade and high-grade prostate tumor samples from the Human Protein Atlas. Numbers on top right corner of each panel indicates the patient ID number. Scale bar: 100 µm. Image credit: Human Protein Atlas; images available from http://v15.proteinatlas.org/, under accession numbers: CNN1, ENSG00000130176; ACTA2, ENSG00000107796 and VIM, ENSG00000026025, in ‘prostate’ or ‘prostate+cancer’ image files. See Materials and Methods for further details. (J) The mRNA expressions of stromal marker genes (*CNN1*, *ACTA2* and *VIM*) from TCGA RNA-seq data with 330 primary PCa samples (Tumor) and 27 normal controls (Normal). (K) The mRNA expression of *CNN1* in PCa samples graded with Gleason scores from TCGA RNA-seq data. (L) The mRNA expressions of stromal marker genes from MSKCC Prostate Oncogenome Project. *P<0.05; **P<0.01; ****P<0.0001 by Student's unpaired *t*-test. Data are presented as mean±s.e.m. Each data point represents one sample. (M) Kaplan–Meier relapse-free (months) analysis of patient data from PCa samples from MSKCC Prostate Oncogenome Project with *CNN1* low expression (*n*=38) and high expression (*n*=93); *P* value is calculated via Mantel–Cox (log rank) test.

In order to understand the degree to which mouse PCa models reflect the stromal changes seen in human PCa, as the proportions of SM and fibroblasts in the normal human prostate are different from mouse, we first characterized human PCa protein and RNA expression databases for changes in stromal markers. Using actin, alpha 2, smooth muscle, aorta (ACTA2) and calponin (CNN1) as SM markers in an analysis of human PCa specimens in the Human Protein Atlas (http://www.proteinatlas.org; Uhlen et al., 2015), we found the expected large proportion of SM in the normal human prostate, and disruption of the well-organized SM layers in PCa samples, especially more advanced tumors (Fig. 1A-F; Fig. S1, S2). The mature SM marker CNN1 was consistently decreased (Fig. 1A-C; Fig. S1) as previously reported (Tuxhorn et al., 2002), and the area of ACTA2 expression seemed decreased in most, but not all, PCa samples (Fig. 1D-F; Fig. S2). However, the proportions of ACTA2-expressing and non-expressing cells varied both within samples from the same individual and between individuals (Fig. S2H-J,H′-J′); thus, it was difficult to determine if SMCs are depleted in tumors. Using vimentin (VIM) as a fibroblast marker, we found that VIM was maintained in non-epithelial cells and possibly increased in some samples in the remaining stroma between glands (Fig. 1G-I; Fig. S3). Overall, higher-grade tumors seemed to have a greater disruption of the stromal cytoarchitecture (Fig. 1A-I; Fig. S1-S3).

As a complementary approach to analyzing human PCa stromal content, we analyzed RNA-sequencing (RNA-seq) data from The Cancer Genome Atlas (TCGA) dataset (*n*=27 normal and 330 primary PCa samples) (Abeshouse et al., 2015), and found a significant decrease in expression of the SM markers *ACTA2* and *CNN1*, as well as the fibroblast marker *VIM* in PCa compared with normal prostate samples (Fig. 1J). In addition, the expression levels of *CNN1* and *ACTA2* (but not *VIM*) were progressively lower in individuals with higher Gleason scores, with a significant decrease seen in Gleason ≥8 compared with most other stages (Fig. 1K; Fig. S4A). There was the expected corresponding decrease in basal cell markers (*KRT5* and *TP63*) and increase in expression of luminal cytokeratin markers in tumor cells (*KRT8* and *KRT18*) (Fig. S4B). There also was a greater correlation between tumors with high *ACTA2* or *CNN1* expression and basal cell gene expression (Pearson coefficient=0.343 or 0.381 for *ACTA2* or *CNN1* with *KRT5*; =0.299 or 0.319 for *ACTA2* or *CNN1* with *TP63*) than for *VIM* and basal markers (Pearson coefficient=0.144 for *VIM* with *KRT5*; =0.097 *VIM* with *TP63*), and a trend towards a negative correlation for the luminal marker *KRT18* with *ACTA2* and no correlation with *CNN1* (Pearson coefficient=−0.139 or −0.067 for *ACTA2* or *CNN1* with *KRT18*) (Table S1A).

As an additional means to address stromal gene expression levels and clinical outcome, we analyzed RNA microarray data from the Memorial Sloan Kettering Cancer Center (MSKCC) Prostate Oncogenome Project (*n*=29 normal, 131 primary tumors, and 19 metastases) (Taylor et al., 2010). Interestingly, metastatic PCa samples expressed significantly lower levels of *CNN1* and *ACTA2* compared with primary tumor samples and normal (Fig. 1L). Furthermore, Kaplan–Meier analysis revealed that patients with lower expression levels of *CNN1* and *ACTA2* but not *VIM* had a significantly shorter relapse-free time (Fig. 1M; Fig. S4C). Taken together, these results show that more advanced PCa samples with a higher Gleason score or metastasis stage have lower levels of expression of two SM markers (*ACTA2* and *CNN1*), indicating either a reduction in the number of SMCs or the expression of these genes is decreased in the remaining cells.

### *PB-MYC* and *ERG/PTEN* but not *TRAMP* mouse PCa models display extensive disruption of smooth muscle

**Fig. 2.**
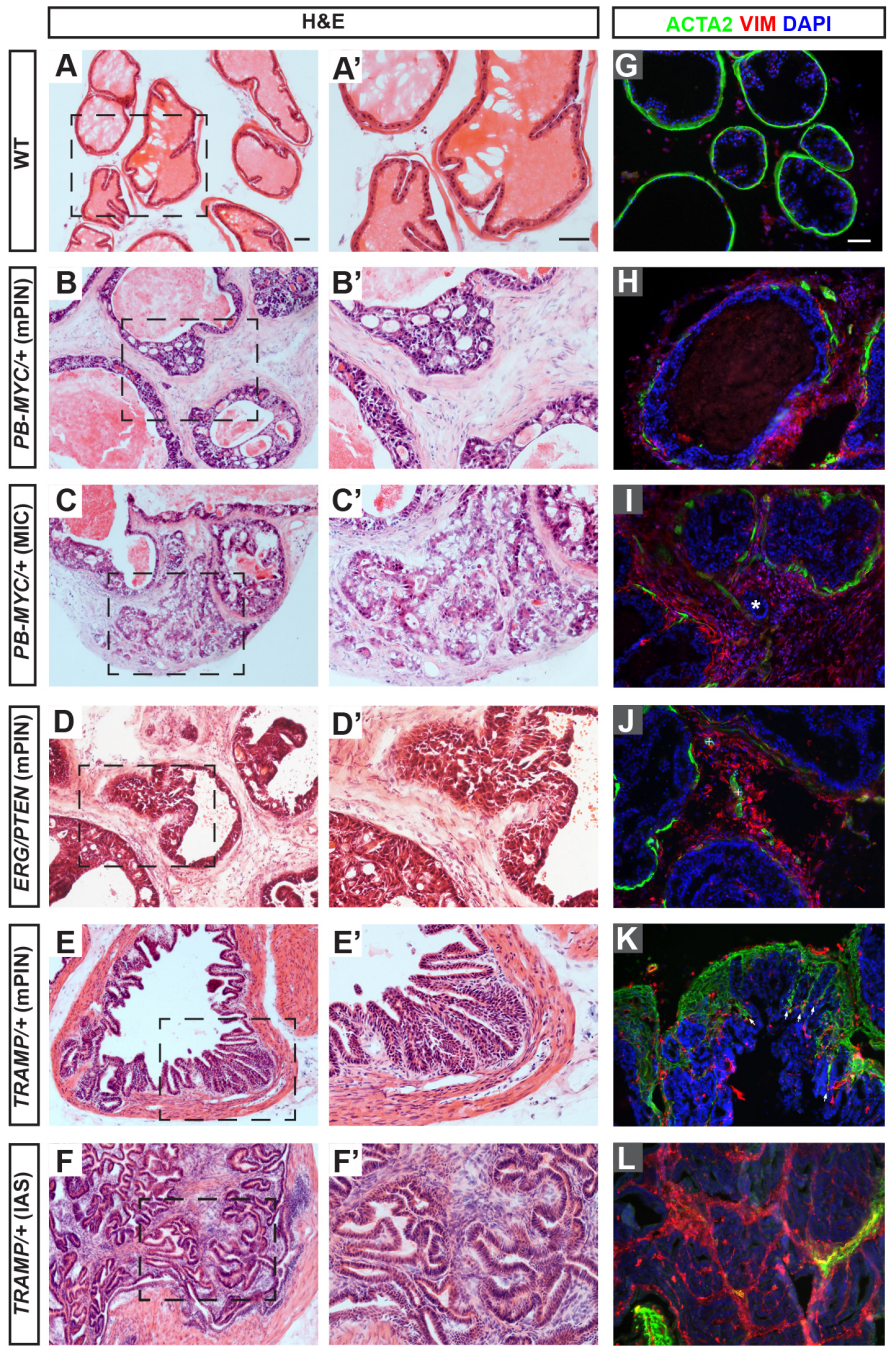
***PB-MYC*, *ERG/PTEN* and *TRAMP* PCa models have distinct stromal characteristics.** (A-F) H&E staining of dorsolateral prostate (DLP) sections from tissues of WT (A), *PB-MYC/+* (B,C), *ERG/PTEN* (D), and *TRAMP/+* (E,F) mice. (A′-F′) Magnification of areas within dashed lines in A-F. (G-L) Immunofluorescence (IF) staining of DLP sections for ACTA2 (green), vimentin (VIM, red), and DAPI (blue). Arrows in K indicate SMCs invading between epithelial folds; * in I indicates infiltrating nests of tumor cells; plus symbols (+) in J indicate blood vessels. Scale bars: 50 µm.

To investigate whether mouse PCa models have similar stromal alterations to those seen in human PCa, especially as mouse normal prostate has less SM than human, we analyzed the stromal characteristics of three distinct mouse lines. Two transgenic lines were investigated that use a probasin gene regulatory element to drive expression of the oncogenes *MYC* (*PB-MYC*) (Ellwood-Yen et al., 2003) or SV40 large/small T antigen (transgenic adenocarcinoma mouse prostate or *TRAMP*) (Greenberg et al., 1995) in the dorsolateral epithelium of mouse prostate, and one conditional genetic model (*ERG/PTEN*) that mimics the *TMPRSS2-ERG* fusion loci that are seen in ∼50% of human PCa (Clark et al., 2007; Hermans et al., 2006) by misexpressing the ETS transcription factor ERG (Chen et al., 2013) combined with *Pten* deletion in luminal cells (*Tmprss2^CreER-GFP/+^; R26^LSL-ERG-GFP/ LSL-ERG-GFP^*; *Pten^flox/flox^* mice administered tamoxifen at 4 weeks of age). Unlike normal mouse prostates with a thin single layer of epithelium (Fig. 2A,A′; Fig. S5A), all three models displayed extensive mPIN, featuring stratified epithelial cells with prominent nuclear atypia forming cribriform and/or tufting confined within the basement membrane. Such multifocal proliferative lesions were found to affect most of the dorsolateral ducts in *PB-MYC* (*n*=52 mice, 35-49 weeks old; Fig. 2B,B′; Fig. S5B) and almost all dorsolateral ducts in *ERG/PTEN* mice (*n*=9, 12-41 weeks old; Fig. 2D,D′; Fig. S5C). In late-stage prostate tumors of *PB-MYC* mice (*n*=8 mice, 44-49 weeks old), micro-invasive carcinoma (MIC) was seen with nests of atypical tumor cells (EpCAM+ and CK5−) infiltrating into the stroma and forming irregular contours (Fig. 2C,C′; Fig. S5B). Using an anti-ACTA2 antibody to label SMCs, we found that the SM layers in *PB-MYC* tumors were much thinner than normal and contained many gaps (Fig. 2G-I; Fig. S5E,F). In *ERG/PTEN* tumors, the disruption of the SM layers was less dramatic; the majority of ducts had an intact SM layer but some ducts had a discontinuous SM layer (Fig. 2J; Fig. S5G). In addition, in contrast to the normal prostate, VIM-positive fibroblast-like cells were abundant in both *PB-MYC* and *ERG/PTEN* prostates, filling up the space between tumor ducts (Fig. 2H-J). Furthermore, the interductal stromal cells showed an increase in expression of collagen 1 (COL1) in *PB-MYC* and to a lesser extent in *ERG/PTEN* tumors (Fig. S6B-B″,C-C″) compared with wild-type (WT) (Fig. S6A-A″). Thus, these two models, and particularly *PB-MYC*, recapitulate many of the stromal alterations reported in human PCa, and therefore are valuable tools to study PCa stromal features and functions.

**Fig. 3.**
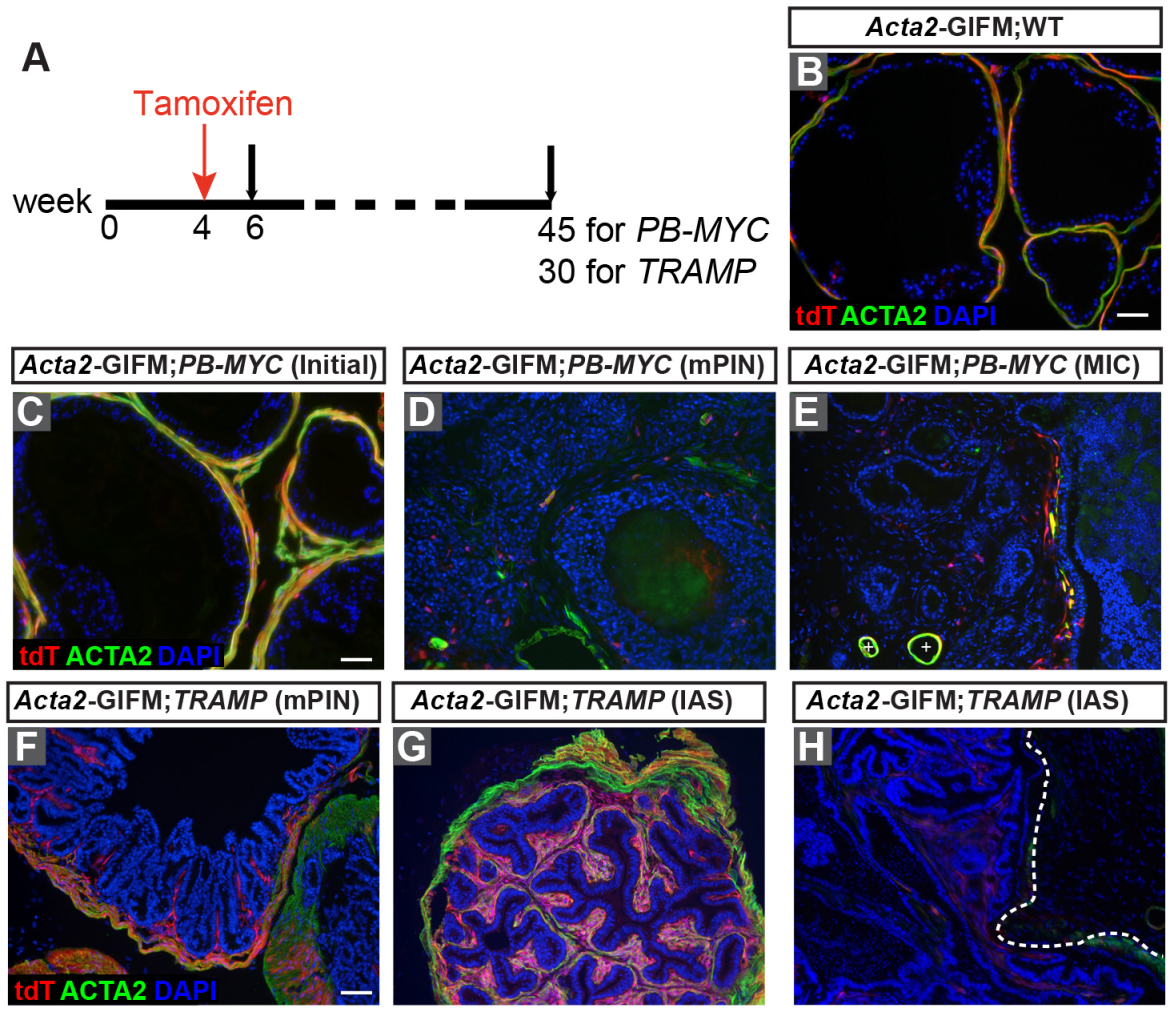
***Acta2*-expressing cells are largely lost in *PB-MYC* tumors whereas some transform into cancer stroma in *TRAMP* IAS lesions.** (A) Schematic showing experimental design. (B) IF staining of DLP sections from 30-week-old *Acta2-CreER/+*;*R26^tdTomato/+^* (*Acta2*-GIFM;WT) mice for tdT (red), ACTA2 (green) and DAPI (blue). (C-E) IF staining of 6-week-old (C) and 45-week-old (D,E) *Acta2-CreER/+*;*R26^tdTomato/+^;PB-MYC/+* (*Acta2*-GIFM;*PB-MYC*) mice for tdT (red), ACTA2 (green) and DAPI (blue). Plus symbols (+) in E indicate blood vessels. (F-H) IF staining of DLP sections from 30-week-old *Acta2*-GIFM;WT (*Acta2-CreER/+;R26^tdTomato/+^*) (F) and *Acta2*-GIFM;*TRAMP* (*Acta2-CreER/+;R26^tdTomato/+^;TRAMP/+*) (G,H) mice for tdTomato (tdT, red), ACTA2 (green), and DAPI (blue). Dashed line in H indicates a region where the SM layer is diminished. Scale bar: 50 µm.

The tumors in *TRAMP* mutants had a distinct stromal character to that in *PB-MYC* and *ERG/PTEN* mice, even when the neuroendocrine tumors were not included in the analysis. In *TRAMP* prostates, most areas with mPIN (*n*=35 mice, 20-33 weeks old; Fig. 2E,E′) showed an increase in the thickness of the SM layer surrounding the ducts (Fig. 2K; Fig. S5H) and ACTA2+ cells invaded between epithelial folds compared with normal prostates (Fig. 2G; Fig. S5E). Interestingly, in more advanced *TRAMP* tumors (28-33 weeks) there were regions with complex polypoid intraductal adenomatous-stromal proliferation with minimal cytologic atypia, referred to as intraductal adenoma with stroma (IAS) as they contained proliferating masses of stromal cells that invaded between the epithelial layers within ducts (*n*=21 mice; Fig. 2F,F′; Fig. S5H). The majority of stromal cells in IAS expressed either ACTA2, VIM, and/or COL1 strongly (Fig. 2L; Fig. S6E-E′), indicating the stromal cells that invaded IAS lesions of *TRAMP* prostates have features of SMCs and/or fibroblasts. Also, unlike in the *PB-MYC* and *ERG/PTEN* models, *TRAMP* tumors had few VIM+ or COL1+ interductal fibroblasts (Fig. S6D). The exception was in rare areas where the SM layer was partially disrupted (Fig. 3H). Thus, only in some areas of *TRAMP* tumors did the stromal character have similarities to the *PB-MYC* and *ERG/PTEN* models. Unlike human PCa, however, in all three PCa models CNN1 expression largely overlapped with ACTA2 expression (Fig. S5I-L).

### SMCs are largely lost in *PB-MYC* prostate tumors whereas some form the stroma in *TRAMP* IAS lesions

As SM is significantly reduced in *PB-MYC* tumors, we used GIFM to test whether ACTA2+ cells are lost or change fate into cancer-reactive stroma. Tamoxifen was administered to *Acta2-CreER/+*;*R26^tdTomato/+^;PB-MYC/+* (*Acta2*-GIFM;*PB-MYC*) mice and control *Acta2-CreER/+*;*R26^tdTomato/+^* (*Acta2*-GIFM;WT) littermates at 4 weeks of age (Fig. 3A). In prostates of *Acta2*-GIFM;*PB-MYC* mice at 6 weeks of age, soon after the initiation of *MYC* transgene expression and two weeks after administration of tamoxifen, the histology and distribution of tdT+ cells was comparable to that of control *Acta2*-GIFM;WT mice (Fig. 3B), and tdT specifically labeled the majority of SMCs (Fig. 3C). Strikingly, *Acta2*-GIFM;*PB-MYC* mice at 45 weeks had a huge reduction in the number of tdT-labeled ACTA2+/COL1+ cells surrounding the mPIN or MIC lesions (Fig. 3D,E; Fig. S7). There were only rare tdT+ reactive stromal cells interspersed between ducts, and they all expressed VIM+ and a rare subset expressed ACTA2 weakly (Fig. S7A-A″,B-B″). This result indicates that ACTA2-positive lineage cells were prominently reduced in number during tumor progression, and they are not a major cell-of-origin for reactive stroma in *PB-MYC* tumors.

As *TRAMP* tumors have a large increase in SM, and stromal cells invade into IASs, we also fate-mapped the *Acta2*-lineage cells in *TRAMP* mice to test whether they expand only in the SM layer or give rise to other cancer reactive stroma. The increased SM layers surrounding mPIN and most IASs remained largely positive for tdT and ACTA2 (Fig. 3F,G). In addition to expanding the SM, some cells marked by *Acta2*-GIFM (many positive for COL1 and negative or only weakly positive for ACTA2) extended into subepithelial folds of mPIN lesions (Fig. 3F) and were present within IASs (Fig. 3G; Fig. S8A-A″,E-E″). Of the intraductal masses, 71% (15/21 from five mice) had extensive tdT labeling (Fig. S8A-A″,B; Table S2), whereas 29% did not (Fig. S8C,D), indicating that SMCs are a major cell-of-origin of the stromal cells within IASs in *TRAMP* tumors. However, none of the fibroblasts between the ducts were labeled with tdT, indicating that as in *PB-MYC* tumors, the interductal cancer stromal cells have a separate cell-of-origin. In addition, in rare regions where the SM layer was diminished there were few fate-mapped cells (Fig. 3H). Among the tdT+ IASs, 10/15 expressed ACTA2 (Table S2), suggesting that some ACTA2-expressing cells turned off ACTA2 while giving rise to cancer reactive stroma. In summary, our two fate-mapping studies indicate that the character of the epithelial tumor cells could regulate the fate of SMCs in prostate tumor stroma.

### *Gli1* is increased and restricted to stromal cells in PCa

**Fig. 4.**
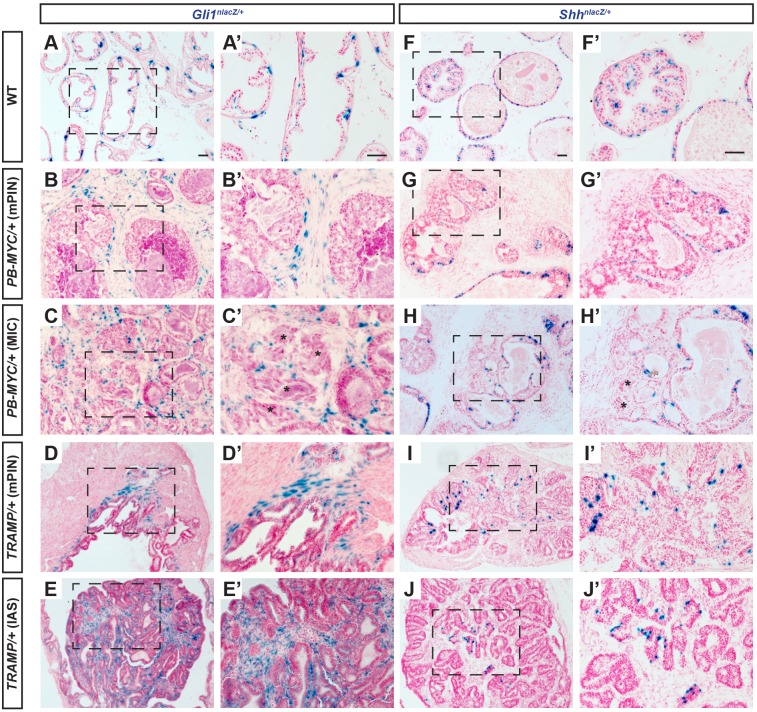
***Gli1^nlacZ^* is expressed in the stromal cells and *Shh^nlacZ^* is expressed in the basal epithelial cells in both *PB-MYC* ­and *TRAMP* prostate tumors.** (A-E) X-GAL staining (blue) of DLP sections from tissues of *Gli1^nlacZ/+^* (A), *Gli1^nlacZ/+^;PB-MYC/+* (B,C) and *Gli1^nlacZ/+^;TRAMP/+* (D,E) mice. (A′-E′) Magnification of areas within dashed lines in A-E. (F-J) X-GAL staining (blue) of DLP sections from tissues of *Shh^nlacZ/+^* (F), *Shh^nlacZ/+^;PB-MYC/+* (G,H), and *Shh^nlacZ/+^;TRAMP/+* (I,J) mice. (F′-J′) Magnification of areas of dashed lines in F-J. * in C′, infiltrating nests of tumor cells. Scale bars: 50 µm.

Given the correlation between reduced SM layers and higher-grade tumors and functional relationship in the prostate between SHH and expansion of the stroma during development, we next asked whether HH signaling is altered in mouse PCa compared with the normal adult prostate. Our previous study using knock-in reporter mice revealed that *Gli1^nlacZ^* (Bai et al., 2002) is expressed by a subset of all four stromal subtypes in normal adult mouse prostate (Peng et al., 2013) (Fig. 4A,A′; Fig. S9A). Unlike previous studies using RNA *in situ* hybridization or questionable antibodies to analyze human tumor samples (Fan et al., 2004; Sanchez et al., 2004), the nuclear-localized β-GAL protein (encoded by the *lacZ* gene) allows the cell type expressing *Gli1* to be unambiguously identified. In areas of mPIN (*n*=12 mice, 35-49 weeks; Fig. 4B,B′; Fig. S9B) or MIC (*n*=6 mice, 45-49 weeks; Fig. 4C,C′; Fig. S9C) of *PB-MYC* tumors, *Gli1^nlacZ^* was expressed in scattered cells in the stroma, primarily adjacent to the tumor epithelium. As in WT mice, all *Gli1*+ cells were negative for the epithelial marker EpCAM (Fig. S9A-C), demonstrating that *Gli1+* cells are stromal cells. We also examined *Gli1* expression in the prostate stroma of *ERG/PTEN* mice carrying a *Gli1^GFP^* allele (referred to as *Gli1^GFP^;ERG/PTEN*). Although almost all epithelial cells express a high level of nuclear-localized GFP (ERG-GFP and CreER-GFP), any stromal GFP+ cells should reflect expression of *Gli1* because *ERG/PTEN* tumors alone have no GFP+ stromal cells (*n*=5 mice, 12-41 weeks; Fig. S9F-F″). As in *PB-MYC* tumors, a high proportion of *ERG/PTEN* tumor stromal cells near mPIN lesions expressed *Gli1^GFP^*, including SMCs (Fig. S9G-G″).

In *TRAMP* mPIN lesions, *Gli1^nlacZ^* expression was enriched in the stromal cells closest to the tumor epithelium, including the SMCs cells invading between epithelial folds (*n*=8 mice, 25-33 weeks; Fig. 4D,D′; Fig. S9D). Strikingly, many tumor stromal cells in IASs expressed *Gli1^nlacZ^*, although cells at a distance from tumor cells did not (*n*=4 mice, 28-33 weeks; Fig. 4E,E′; Fig. S9E). Thus, an increase in HH signaling specifically in the stroma (SMCs and fibroblasts) near tumor cells seems to be a consistent finding in mouse models of PCa.

### IHH and DHH contribute to HH signaling to the stroma of mouse PCa

We next tested whether SHH could be the ligand responsible for *Gli1* expression near tumor cells by examining *Shh^nlacZ^* (Gonzalez-Reyes et al., 2012) expression in *PB-MYC* and *TRAMP* tumors. As in WT prostate (Peng et al., 2013) (Fig. 4F,F′; Fig. S10A,F), *Shh^nlacZ^* expression was detected in the majority of CK5+ basal cells in *PB-MYC* mPIN lesions, although a few cells were only positive for either CK5 or *Shh^nlacZ^* (*n*=6 mice, 35-42 weeks; Fig. 4G,G′; Fig. S10B,G). In areas of MICs, *Shh^nlacZ^*-expressing cells were rare (*n*=5 mice, 35-42 weeks; Fig. 4H,H′), consistent with the loss of cells with a basal phenotype, as indicated by few CK5-expressing cells (Fig. S10C,H). Similarly, in *TRAMP* mPIN lesions, *Shh^lacZ^* expression was detected in CK5+ basal cells, (*n*=5 mice, 20-32 weeks; Fig. 4I,I′). The number of basal cells, however, was greatly diluted out by the increase in CK8+ luminal cells (Fig. S10D,I). In IASs, *Shh^lacZ^* expression was detected only in the rare CK5+ basal cells that remained (*n*=5 mice, 20-32 weeks, Fig. 4J,J′; Fig. S10E,J).

**Fig. 5.**
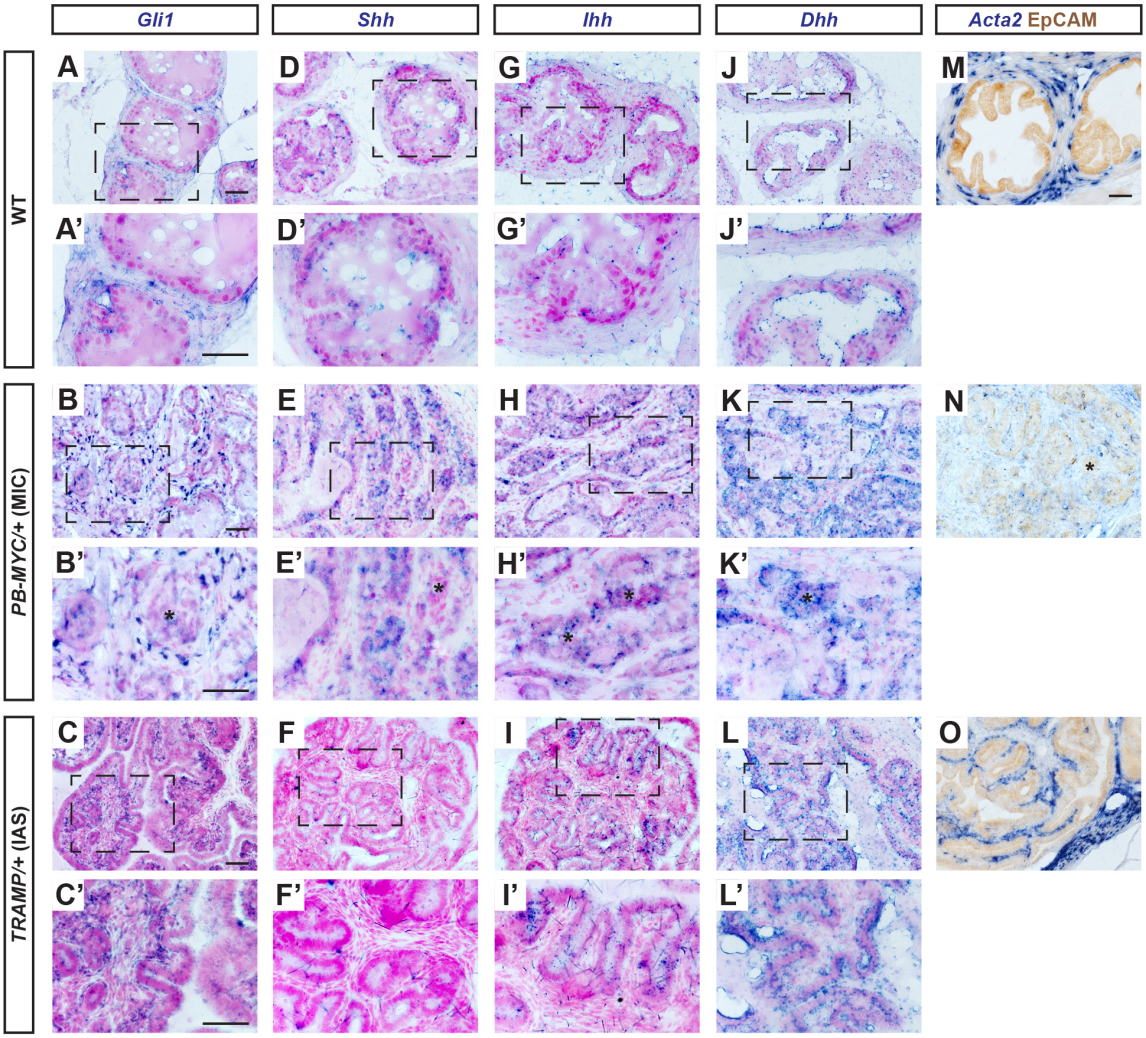
***Ihh* and *Dhh* are upregulated in tumor epithelium of *PB-MYC* and *TRAMP* PCa models.** mRNA *in situ* hybridization (blue) of *Gli1* (A-C), *Shh* (D-F), *Ihh* (G-I), *Dhh* (J-L) on DLP sections from WT (A,D,G,J), *PB-MYC/+* MIC lesions (B,E,H,K), and *TRAMP/+* IAS lesions (C,F,I,L), counter-stained with Fast Red. (A′-L′) Magnifications of areas of dashed lines in A-L. (M-O) *Acta2* mRNA *in situ* hybridization (blue) co-stained with IHC of EpCAM. * indicate infiltrating nests of tumor cells. Scale bars: 50 µm.

Our results from *PB-MYC* and *TRAMP* PCa models suggest that paracrine HH signaling is retained in tumors, but raises the question of whether SHH is the primary ligand as few *Shh^nlacZ^*-expressing cells remained in tumors. One possible explanation for the extensive *Gli1* expression despite little *Shh* in both PCa models is that another HH ligand is expressed. Using *in situ* hybridization (ISH), we first confirmed an increase in cells expressing *Gli1* (Fig. 5A-C,A′-C′; Fig. S11A-C,A′-C′), and also found that *Shh* expression was maintained in *PB-MYC* tumors (Fig. 5E,E′; Fig. S11E,E′) but almost absent in *TRAMP* tumors (Fig. 5F,F′; Fig. S11F,F′) compared with WT (Fig. 5D,D′; Fig. S11D,D′). As expected, *Acta2* mRNA in stromal cells was markedly decreased in *PB-MYC* tumors (Fig. 5N; Fig. S9N) and increased in *TRAMP* tumors (Fig. 5O; Fig. S11O) compared with WT (Fig. 5M; Fig. S11M). Interestingly, whereas we detected little expression of *Ihh* or *Dhh* in the epithelium of normal prostates (Fig. 5G,G′,J,J′), *Ihh* was abundant in *PB-MYC* (Fig. 5H,H′; Fig. S11H,H′) and somewhat increased in *TRAMP* (Fig. 5I,I′; Fig. S11I,I′) tumor epithelium, and *Dhh* was detected in the tumor epithelium of both *PB-MYC* and *TRAMP* models, especially in *TRAMP* mPIN lesions (Fig. 5K,K′,L,L′; Fig. S11 K,K′,L,L′). qRT-PCR analysis of RNA isolated from whole dorsal prostates of WT, *TRAMP* and *PB-MYC* tumors confirmed that *Ihh* expression is highest in *PB-MYC* and *Dhh* is highest in *TRAMP* tumors, with both increased compared with normal prostate (Fig. S12). In summary, our results have uncovered that *Ihh* and *Dhh* expression are increased in the epithelium of two mouse PCa models as compared with normal prostates, with *Ihh* increased more in *PB-MYC* than *TRAMP* tumors.

### HH signaling in human PCa correlates with stromal content and is driven mainly by *DHH*

Our expression studies using mouse tumor sections raise the question of whether the genes encoding any of the HH ligands and *GLI1* are increased in a subset of human PCa. Analysis of the TCGA dataset (*n*=330) of primary PCa samples (Abeshouse et al., 2015) and the MSKCC Prostate Oncogenome Project (*n*=29 normal, 131 primary tumors, and 19 metastases) (Taylor et al., 2010) revealed that the overall expression of *IHH*, but not *SHH* or *DHH* is significantly increased in tumor samples compared with normal prostate samples (Fig. S13A). However, there were no significant differences in the expression of HH genes between tumor samples with various Gleason scores (Fig. S13B) or between primary and metastasis tumors (Fig. S13C). *GLI1* expression was the same in normal prostate and all types of tumor samples (Fig. S13), which could indicate an actual increase in stromal expression as the proportion of stromal cells is reduced. Indeed, there was a positive correlation between tumors with high expression of stromal markers and *GLI1* (Pearson coefficient=0.483, 0.379 or 0.595 for *ACTA2*, *CNN1* or *VIM* with *GLI1*; Table S1B). There was also a correlation between *DHH* expression, and to a lesser extent *IHH* or *SHH*, with *GLI1* expression levels (Pearson coefficient=0.309, 0.198 or 0.181 for *DHH*, *IHH* or *SHH* with *GLI1*; Table S1B). There was also a strong correlation between *DHH* and *ACTA* or *VIM* (Pearson coefficient=0.422 or 0.560 for *ACTA2* or *VIM* with *DHH*). Curiously, there were poor correlations between the levels of *DHH* and luminal markers (Pearson coefficient=0.025 or −0.054 for *KRT8* or *KRT18* with *DHH*) or basal markers (Pearson coefficient=0.001 or −0.017 for *KRT5* or *TP63* with *DHH*) in tumor samples (Table S1B), indicating that *DHH* expression is induced in a tumor context-dependent manner. Thus, mRNA expression data from human primary PCa samples indicate that the level of HH signaling (*GLI1* expression) correlates with the proportion of stromal cells in a tumor, and *IHH* is increased more than the other HH ligands in tumors compared with normal prostate.

### Gene expression profile of *Gli1*-expressing stromal cells is altered in *PB-MYC* tumors

In order to analyze the expression profile of mouse PCa stromal cells, we used the *Gli1^GFP/+^* knock-in reporter line (Brownell et al., 2011) to isolate the subset of stromal cells undergoing HH signaling to compare the transcriptomes between *PB-MYC* PCa and normal prostate (∼45 weeks of age) using RNA-seq. Unsupervised hierarchical clustering of all genes with significant differences in gene expression showed a clear separation between the two populations (Fig. S14A). Analysis of variance correcting for multiple hypothesis testing identified 243 genes with significantly different expression (*P*<0.05 and FDR<0.05) between WT and *PB-MYC* cells expressing *Gli1^GFP^* at a level of ≥twofold (281 genes ≥1.5-fold) (Table S3A), with the majority of the genes expressed more strongly in WT cells (Fig. S14A). Interestingly, *Acta2* and *Cnn1* were decreased by 21- and 12-fold, respectively, in stromal *Gli1*-expressing tumor cells compared with WT, and VIM was not significantly altered (Table S3B). The decrease in SM gene expression likely reflects the decrease in the proportion of SMCs in *PB-MYC* tumors. Consistent with our conclusions based on RNA *in situ* analysis, the three *Hh* genes were not expressed in stromal *Gli1*-expressing cells (Table S3B). Pathway analysis (*P*<0.1 and FDR<0.1) identified six pathway differences (Fig. S14B), with a top pathway being focal adhesion that was reduced in *PB-MYC Gli1^GFP^*-expressing cells (Fig. S14B,C), consistent with the greater dispersion of stromal cells in tumors.

### Enhanced HH signaling in the *PB-MYC* stroma impedes PCa progression and maintains the SM

**Fig. 6.**
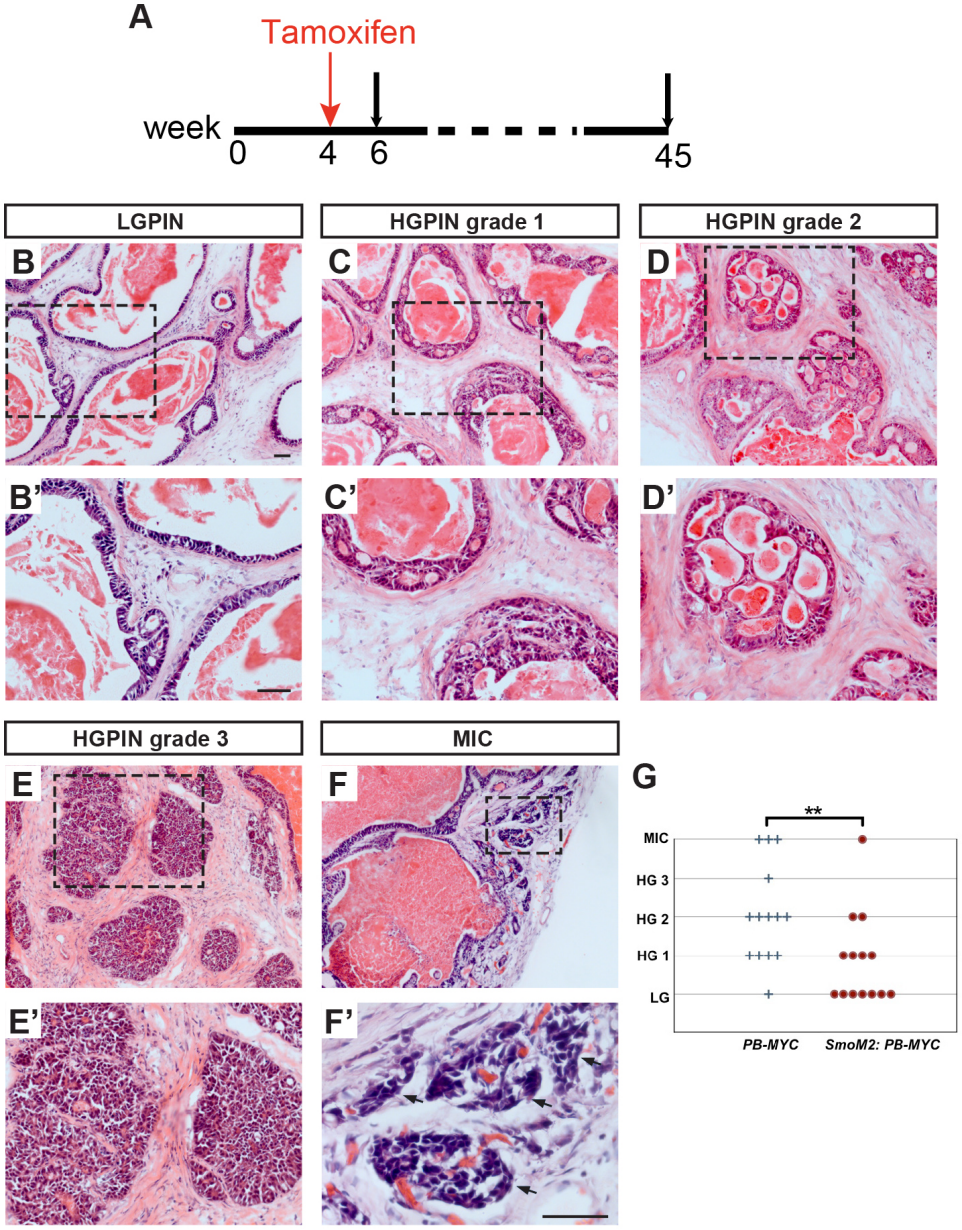
**Ectopic HH signaling in the stroma reduces *PB-MYC* prostate tumor progression.** (A) Schematic showing experimental design. 45 indicates 44-46 weeks. (B-F) H&E staining of DLP sections from tissues of either *SmoM2;PB-MYC* (*Gli1^CreER/+^;R26^LSL-SmoM2-YFP/+^;PB-MYC/+*) or *PB-MYC* (*R26^LSL-SmoM2-YFP/+^;PB-MYC/+*) mice, representing lesions of different severity; low-grade mPIN (LGPIN or LG) (B), high-grade 1 mPIN (HGPIN grade 1 or HG 1) (C), high-grade 2 mPIN (HGPIN grade 2 or HG 2) (D), high-grade 3 mPIN (HGPIN grade 3 or HG 3) (E), and MIC (F). (B′-F′) Magnification of areas of dashed lines in B-F. Arrows indicate micro-invasive carcinoma. Scale bars, 50 µm. (G) Dot plot of each mouse with a certain grade of tumor lesion, showing inverse correlation between tumor severity and HH signaling. Each dot represents one mouse. **P<0.01 by Mann–Whitney U test; *n*=14 mice for each of *SmoM2;PB-MYC* and *PB-MYC* groups.

Given the extensive reduction of SM and presence of local MIC in advanced *PB-MYC* tumors, and reduction in SM gene expression in more advanced human PCa samples, we reasoned that the damaged SM layers could facilitate the invasion of prostate tumor cells, as they must cross the SM layer surrounding the ductal glands. Furthermore, given the correlation between high *DHH* and *GLI1* expression and higher stromal content of human PCa samples, we hypothesized that increasing stromal HH signaling would decrease tumor progression by maintaining or increasing SM. To test this hypothesis, we used Cre/loxP to genetically increase HH signaling in the stroma of *PB-MYC* tumors by administering tamoxifen to *Gli1^CreER/+^;R26^LSL-SmoM2-YFP/+^;PB-MYC/+* (*SmoM2;PB-MYC*) and *R26^LSL-SmoM2-YFP/+^;PB-MYC/+* (*PB-MYC*) littermates at 4 weeks to induce expression of a constitutively active SMO (SmoM2) (Mao et al., 2006) in a subset of stromal cells (Fig. 6A). The severity of tumors was assessed at 44 to 46 weeks in a blinded fashion based on tissue pathology. Four categories of mPIN lesions and MIC were graded according to the degree of architectural and cytological abnormalities and the extent of ducts affected. Low-grade mPIN (LGPIN) was defined as having one to two layers of cells and mild nuclear atypia (Fig. 6B,B′). High-grade mPIN lesions were divided into three grades: HGPIN1 lesions were focal and had increased nuclear atypia with two or more layers of cells often in papillary, tufting, or cribriform arrangements (Fig. 6C,C′). HGPIN2 lesions were more extensive and had obvious nuclear atypia and the cells filled or almost filled the ductal lumens in papillary or cribriform patterns (Fig. 6D,D′). HGPIN3 lesions were the most extensive and had more severe atypia, filled the ductal lumens, and some cells bulged into the surrounding stroma but without the clear invasion seen in MIC (Fig. 6E,E′). Strikingly, mice with active HH signaling in the stroma (*SmoM2;PB-MYC*) showed a significant decrease in tumor grade compared with *PB-MYC* littermates (Fig. 6G) (Mann–Whitney U test: *U*=53, *n_1_*=*n_2_*=14, *P*=0.0096 two-tailed). Thus, increased HH signaling in *PB-MYC* stroma can suppress progression of PCa.

**Fig. 7.**
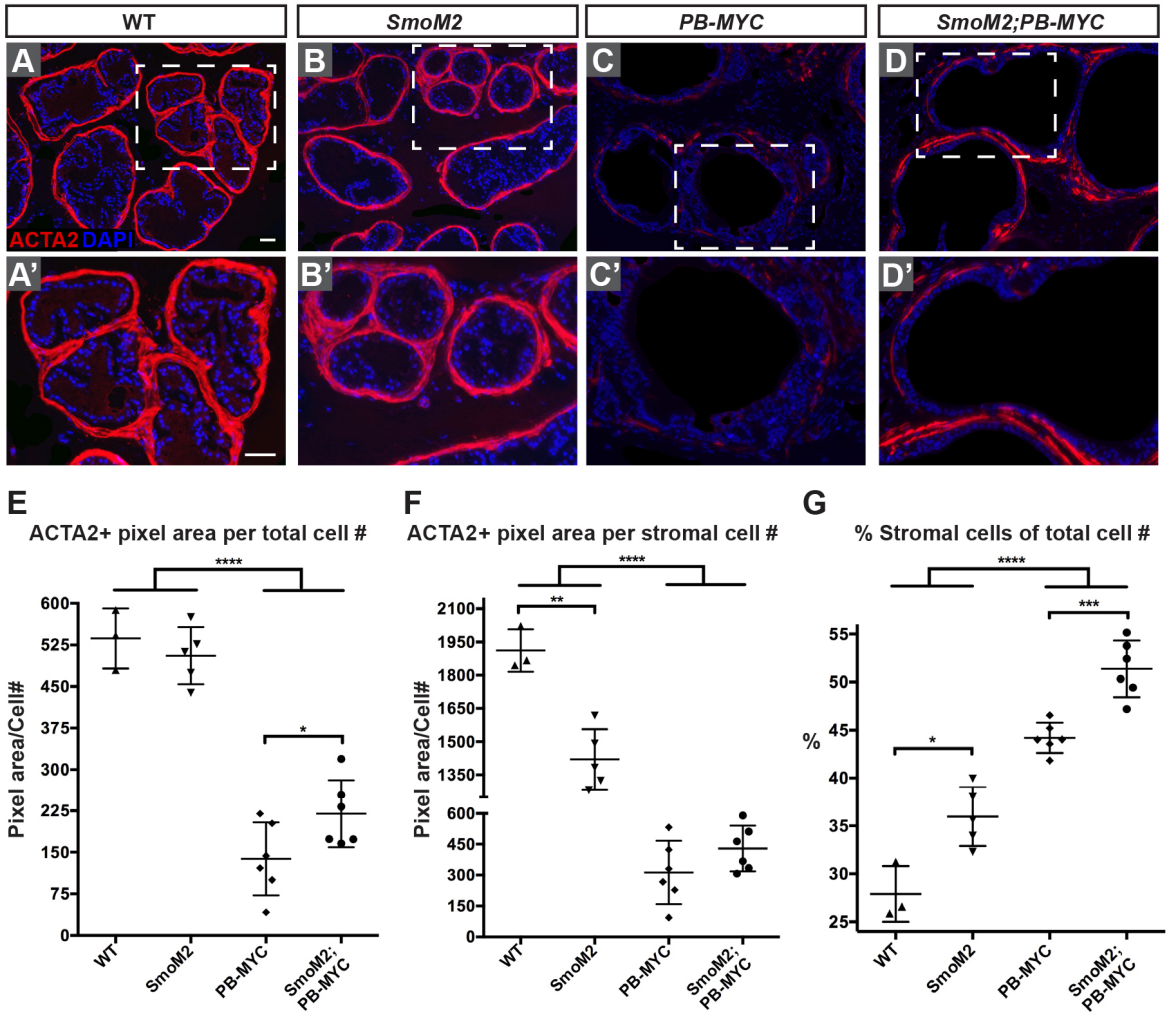
**Enhanced HH signaling in the stroma of *PB-MYC* prostates increases stromal cells including SMCs.** (A-D) IF staining of DLP sections from WT (*R26^LSL-SmoM2-YFP/+^*) (A), SmoM2 (*Gli1^CreER/+^; R26^LSL-SmoM2-YFP/+^*) (B), *PB-MYC* (*R26^LSL-SmoM2-YFP/+^; PB-MYC/+*) (C), and *SmoM2;PB-MYC* (*Gli1^CreER/+^; R26^LSL-SmoM2-YFP/+^; PB-MYC/+*) (D) mice for ACTA2 (red) and DAPI (blue). (A′-D′) Magnification of areas of dashed lines in A-D. Scale bars: 50 µm. (E-F) Quantification of the ACTA2+ area per total cell number (E) or per stromal cell number (F). (G) The percentage of stromal cells within total cells. *P<0.05; **P<0.01; ***P<0.001; ****P<0.0001 by Student's unpaired *t*-test. Data are presented as mean±s.e.m. Each data point represents the average of four sections from one mouse.

As MIC is less likely to occur in *PB-MYC* tumors with increased stromal HH signaling (Fig. 4F), we then asked whether the SMC content of tumors was altered by HH activation. The ACTA2+ area was quantified in *SmoM2;PB-MYC* mice and *PB-MYC* littermates (*n*=6 mice, 44-45 weeks), as well as their non-tumor littermate controls, *Gli1^CreER/+^;R26^LSL-SmoM2-YFP/+^* (SmoM2) (*n*=5 mice, 45 weeks) and *R26^LSL-SmoM2-YFP/+^* (WT) (*n*=3 mice, 45 weeks) mice. Whereas *PB-MYC* tumors had the expected large decrease in SMCs and gaps in the SM layer compared with normal prostates (Fig. 7A,A′,C,C′), *PB-MYC* tumors with stromal SmoM2 expression had strikingly more normal SM layers (Fig. 7C,C′,D,D′). Quantification of the ACTA2+ area with respect to total cell number (estimated from the number of DAPI+ nuclei) showed an increase (*P*=0.049) in *SmoM2;PB-MYC* mice compared with *PB-MYC* mice (Fig. 7E), although ACTA2+ area relative to stromal cell number (EpCAM–) did not show a significant increase (Fig. 7F). This result could in part be due to a contribution of immune cells to the stromal cell count. The increase of SM in *SmoM2;PB-MYC* tumors is unlikely to result from enhanced HH signaling via *SmoM2* expression as the SMCs are not specifically enriched with SmoM2-YFP-positive cells, and both ACTA2+ and ACTA2– stromal cells expressed SmoM2-YFP (Fig. S15). These results suggest that HH signaling maintains the SM layers in PCa and has an inhibitory effect on invasive cancer, possibly via maintaining the SM layers as barriers to prevent tumor epithelial cells from invading into the stroma.

### Enhanced HH signaling in the stroma of *PB-MYC* tumors increases the stromal cell number

Although *PB-MYC* tumors have a prominent reduction of SMCs, the proportion of all cells (epithelial+stromal) that were stromal (EpCAM–) was significantly increased in both *SmoM2;PB-MYC* (*P*<0.0001) and *PB-MYC* (*P*<0.0001) prostates compared with non-tumor controls, and was significantly higher in *SmoM2;PB-MYC* than *PB-MYC* tumors (*P*=0.0004) (Fig. 7G). There was also a small increase in the proportion of stromal cells in *SmoM2* non-tumor prostates compared with WT (*P*=0.01) (Fig. 7G). These results indicate that the major effect of increasing HH signaling in the stroma of *PB-MYC* tumors is to increase the proportion of stromal cells.

**Fig. 8.**
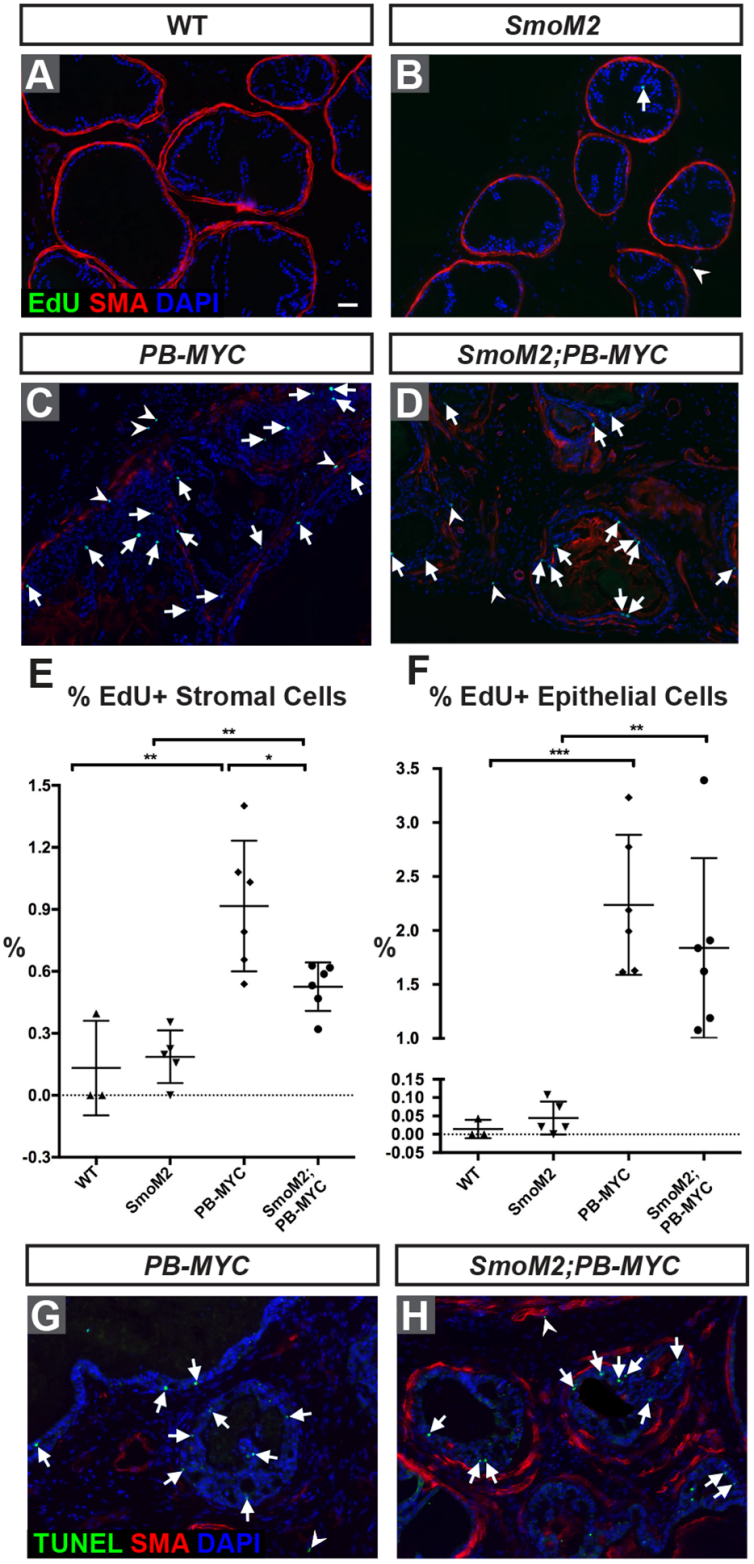
**Enhanced stromal HH signaling in *PB-MYC* prostates does not increase stromal cell proliferation in late stage tumors.** (A-D) IF staining of DLP sections from WT (A), SmoM2 (B), *PB-MYC* (C), and *SmoM2;PB-MYC* (D) mice for EdU (green), ACTA2 (red) and DAPI (blue). Scale bar: 50 µm. Arrows indicate EdU+ epithelial cells; arrowheads indicate EdU+ stromal cells. (E,F) Quantification of the percentage of EdU+ stromal cells (E) or EdU+ epithelial cells (F). *P<0.05; **P<0.01; ***P<0.001 by Student's unpaired *t*-test. Data presented as mean±s.e.m. (G-H) TUNEL staining (green) with ACTA2 (red) and DAPI (blue) of DLP sections from *PB-MYC* (G) and *SmoM2;PB-MYC* (H) mice. Arrows indicate TUNEL+ epithelial cells; arrowheads indicate TUNEL+ stromal cells.

The observed increase in stromal cells in *SmoM2;PB-MYC* prostates could result from an increase in proliferation (1 h pulse EdU) or a decrease in cell death induced by HH activation. In *SmoM2* and WT prostates, few EdU+ cells were detected (Fig. 8A,B,E,F), whereas *SmoM2;PB-MYC* and *PB-MYC* tumors had the expected significantly higher level of cell proliferation (*P*=0.001 and *P*=0.0007, respectively). Curiously, the percentage of EdU+ stromal cells was significantly reduced (*P*=0.018) in the stroma of *SmoM2;PB-MYC* mice compared with *PB-MYC* in late stage tumors (Fig. 8C,D,E), and in epithelial cells was slightly but not significantly (*P*=0.37) lower (Fig. 8F). TUNEL labeling of dying cells did not reveal an obvious difference between *SmoM2;PB-MYC* and *PB-MYC* tumors (Fig. 8G,H). Thus, the main cellular changes that lead to the increase in PCa stroma likely occur at an earlier stage in tumor progression, or because there are more stromal cells in *PB-MYC* tumors when HH signaling is increased.

## DISCUSSION

In three mouse models of PCa, including one with highly recurrent mutations in human PCa (*ERG/PTEN*), we found distinct contributions of SM-like and fibroblast-like cells to the stroma, yet HH signaling (*Gli1^nlacZ^* expression) was restricted to stromal cells, especially near the tumor epithelium. Whereas *TRAMP* tumors had an increase in SMCs and little change in interductal fibroblasts, *PB-MYC*, and to a lesser extent *ERG/PTEN*, had a decrease in SMCs and an accompanying increase in fibroblast-like stromal cells between the ducts. RNA-seq analysis of *Gli1^GFP^*-labeled cells of *PB-MYC* and normal prostate similarly revealed a major decrease in SM genes, and the fibroblast gene *Vim* was not altered. Based on the Human Protein Atlas and RNA data analysis, we confirmed that human PCa tumors have a dramatic disruption of the SM layers and a clear decrease in the mature SM marker calponin (CNN1) in advanced tumors, consistent with a previous report (Tuxhorn et al., 2002). ACTA2 staining revealed the SM layers were disrupted and the proportion of cells was decreased in most advanced tumor samples, whereas VIM was maintained in the remaining stromal cells. Analysis of TCGA, the largest RNA-seq dataset of primary PCa samples (Abeshouse et al., 2015), revealed a decrease in *ACTA2* and *CNN1* but not *VIM* expression in more advanced tumors (higher Gleason score), suggesting the proportion of SMCs in human tumors, or at least the expression levels of the cell type-specific genes are decreased. In a separate RNA expression dataset (Taylor et al., 2010) *ACTA2* and *CNN1* but not *VIM* expression were found to be significantly lower in metastatic samples compared with primary tumors. In addition, we found correlations between higher luminal or lower basal cell gene expression and lower SM gene expression. Our study thus demonstrates that PCa in *PB-MYC* mice nicely models the decrease in SM layers seen in more advanced human PCa.

Using GIFM, we traced the fate of ACTA2-expressing cells *in vivo* during mouse tumor progression and found that the labeled cells are largely lost in *PB-MYC* tumors without changing fate and giving rise to cancer reactive stroma. In *TRAMP* tumors, in contrast, labeled SMCs not only expand the SM layers, but also contribute to cancer stromal cells, specifically in IAS and not between ducts. Thus, in *TRAMP* tumors, some labeled SMCs change their fate to fibroblasts and/or myofibroblast-like cells and migrate into IASs. However, SMCs in *TRAMP* tumors, as in *PB-MYC* tumors, do not contribute to interductal stromal cells. We propose that the fate of SMC is likely determined by the molecular character of the tumor epithelial cells. It will be interesting to determine if the cell-of-origin of interductal stroma is the stem or progenitor cell that a fate-mapping study indicates is restricted to the interductal fibroblast lineage (Peng et al., 2013). Furthermore, if each stromal lineage has a distinct expression signature, it should be possible to predict the cell of origin of reactive stroma in human PCa samples.

In both *TRAMP* and *PB-MYC* models of PCa, we found that the proportion of epithelial cells expressing *Shh^nlacZ^* decreases greatly during tumor progression, whereas the two alternate ligands, *Ihh* and *Dhh*, are prominently expressed by tumor cells but not normal prostate luminal cells (Fig. 5; Fig. S9). Consistent with this result, *Shh* expression is decreased and *Ihh* increased in the *LADY* prostate tumor model compared with normal prostate based on qRT-PCR of whole tumor tissue (Gipp et al., 2007; Kasper et al., 1998). Analysis of TCGA RNA-seq data revealed that *IHH* is increased in human PCa compared with normal prostate (Fig. S4). Furthermore, the level of *DHH*, and to a lesser extent *IHH*, is positively correlated with *GLI1* (HH signaling), as well as the level of stromal gene expression (Table S1B). Thus, HH signaling seems to be a predictor of the amount of stroma in a tumor. Finally, our examination of gene expression on mouse sections revealed a paracrine mode of HH signaling from tumor cells to stroma.

A majority of the studies on the role of HH signaling using PCa cell lines have suggested that HH pathway blockade via cyclopamine treatment suppresses tumor growth. Using an *in vivo* genetic mouse model that reflects the changes in stromal content of human PCa, however, we found that aberrant activation of HH signaling in the *Gli1*-expressing subset of stromal cells in *PB-MYC* tumors results in decreased tumor progression, revealing that tumor stroma can restrain PCa progression. We propose that the partially restored SM layers act as a barrier to prevent epithelial cells from invading into the stroma. It is also possible that SMCs secrete factors, such as pro-differentiation proteins, that restrain tumor progression. Together with several recent studies showing that inhibition of HH signaling in the stroma of pancreas and bladder cancers decreases survival (Lee et al., 2014; Rhim et al., 2014; Shin et al., 2014), our findings offer an explanation for the unsuccessful clinical trials using small-molecule HH antagonists for PCa. Further research into genetic models that represent later stages of human PCa will provide additional evidence for the value of altering HH function for PCa patients.

## MATERIALS AND METHODS

### Mice

The following mouse lines were used: *TRAMP/+* (Greenberg et al., 1995), *PB-MYC/+* (Ellwood-Yen et al., 2003), *Shh^nlacZ/+^* (Gonzalez-Reyes et al., 2012), *Gli1^nlacZ/+^* (Bai et al., 2002), *Gli1^GFP/+^* (Brownell et al., 2011), *Gli1^CreER/+^* (Ahn and Joyner, 2005), *Acta2-CreER/+* (also called *Sma-CreER*; Wendling et al., 2009), *Pten^flox/flox^* (Trotman et al., 2003), *R26^LSL-ERG-GFP/ LSL-ERG-GFP^* (Chen et al., 2013), *Rosa26* (*R26*) reporter mice (Madisen et al., 2010; Soriano, 1999; Srinivas et al., 2001), and *Tmprss2^CreER-GFP/+^* generated by knock-in of a *CreER^T2^-IRES-EGFP* cassette with a splice acceptor to replace exon 2 of the *Tmprss2* gene (Gao et al., 2016). Tamoxifen (Sigma, T5648) was dissolved in corn oil and administered by oral gavage (250 μg g^-1^). Mouse husbandry and all experiments were performed in accordance with MSKCC IACUC-approved protocols.

### Tissue processing

Animals were anesthetized and transcardially perfused with PBS followed by chilled 4% paraformaldehyde. Prostates were harvested and postfixed for 15-20 min (normal prostate) or 2-3 h (tumor) or overnight (RNA *in situ* and some immunohistochemistry), and cryoprotected in 30% sucrose before freezing in Cryo-OCT (VWR, 25608-930). Frozen prostates were sectioned at either 8 μm (pathology) or 12 μm on a cryostat, and sections of the dorsolateral prostates were used for all analyses.

### Microscopy

Mosaic fluorescence images were taken on an inverted microscope (Zeiss, Observer.Z1) using Zen software (Zeiss). Bright-field images were taken with 10× or 20× objectives.

### Immunofluorescent staining

Cryosections were stained with the following primary antibodies: anti-ACTA2 (1:500; Sigma-Aldrich, F3777 FITC-conjugated or C6198 Cy3-conjugated), anti-vimentin (1:500; Cell Signaling, 5741), anti-calponin (1:500; Abcam, ab46794), anti-collagen type I alpha 2 (1:500; Rockland, 600-401-103-0.1), anti-β-GAL (1:1000; Thermo Fisher Scientific, PA1-21477), anti-EpCAM (1:200; eBioscience, 14-5791-82), anti-GFP/YFP (1:1000; Nacalai Tesque, 0440484), anti-CK5 (1:2000; Covance, PRB-160P), and anti-CK8 (1:500; Developmental Studies Hybridoma Bank, TROMA-1). Secondary antibodies for double labeling were donkey anti-rabbit IgG (H+L) Alexa Fluor 555 (Invitrogen, A-31572), Alexa Fluor 488 (Invitrogen, A-21206) or Alexa Fluor 647 (Invitrogen, A-31573), and goat anti-rat IgG (H+L) Alexa Fluor 555 (Invitrogen, A-21434), all used at 1:1000. Nuclei were counterstained with DAPI.

### X-GAL staining

Sections were post-fixed with paraformaldehyde for 5 min, washed twice in X-GAL buffer (2 mM MgCl_2_, 0.1% Igepal Ca-30, 0.05% sodium deoxycholate in PBS) for 10 min, and stained in X-GAL staining solution (1 mg ml^−1^ X-GAL, 0.2 mM potassium ferricyanide, 0.17 mM potassium ferrocyanide in X-GAL buffer) for 12-14 h at 37°C. X-GAL-stained sections were counterstained with 0.1% Nuclear Fast Red (Poly Scientific, s248).

### Flow cytometry and RNA-sequencing

To isolate HH-responding (*Gli1*-expressing) stromal cells, prostates of *Gli1^GFP/+^; PB-MYC/+* and *Gli1^GFP/+^* mice were freshly harvested and processed into a single cell suspension, and then subject to fluorescence-activated cell sorting (FACS ) to isolate GFP+ cells. RNA was extracted from GFP+ cells from individual prostates; RNA was then pooled to have a minimum of 4 ng and subject to RNA-sequencing analysis (MSKCC Genomics Core Facility). Alignment of raw data, principal component analysis, and unsupervised hierarchical clustering were performed using Partek Flow software, version 5.0 (Partek Inc., St. Louis, MO, USA). Pathway analysis was performed in DAVID (Huang da et al., 2009a; Huang da et al., 2009b).

### Quantification and statistical analysis

To quantify the area of cells expressing ACTA2, 20× mosaic photographs of four region-matched dorsolateral prostate sections were taken from each male mouse, and the ACTA2+ pixel area was measured using Photoshop (Adobe). The numbers of DAPI+ nuclei in the epithelium (EpCAM+) and stroma (EpCAM−) were measured using Cell Profiler (Jones et al., 2008). To quantify the EdU+ cells in each compartment, 20× mosaic photographs of four region-matched dorsolateral prostate sections were taken from each male, and EdU+/EpCAM+ and EdU+/EpCAM– cells were counted manually using Stereo Investigator (MBF Bioscience). At least three mice were analyzed for each group in each experiment. Data are presented as mean±s.e.m (standard error of the mean). Statistical analyses were performed using GraphPad Prism version 6.0.

### EdU (5-ethynyl-2′-deoxyuridine) injection and staining

For assessing cell proliferation, EdU (Invitrogen, E10187) was given at 100 mg g^−1^ by intraperitonal injection 1 h before euthanasia. Click-it EdU assay with Alexa Fluor 488 (Invitrogen, C10337) was used according to the protocol of the manufacturer.

### TUNEL staining

For TUNEL staining, slides were permeabilized with 0.5% Triton X-100, pre-incubated with Tdt buffer (30 mM Tris HCl, 140 mM sodium cacodylate and 1 mM CoCl_2_) for 15 min at room temperature, and incubated for 1 h at 37°C in TUNEL reaction solution (Tdt buffer containing TUNEL enzyme and dUTPbiotin; Roche Applied Science). Then slides were incubated with Streptavidin Alexa Fluor 647 (Invitrogen, S-32357) for 1 h.

### mRNA *in situ* hybridization

RNA *in situ* hybridization analysis was performed based on standard protocols (Birren et al., 1993; Keil et al., 2012) with minor modifications, using antisense RNA probes for *Shh*, *Dhh* and *Ihh* (Echelard et al., 1993), *Gli1* (Hui et al., 1994), and an *Acta2* probe made using RT-PCR and the following primers: 5′-TGG CTT CGC TGT CTA CCT TC-3′ and 5′-CGA TGT TAA TAC GAC TCA CTA TAG GGT GAA GTC AGT GTC GAT TTT TCC-3′.

### RNA isolation and real-time polymerase chain reaction (qRT-PCR)

Total RNA from dorsal prostates was isolated using miRNeasy mini kit (QIAGEN, 217004). For reverse transcription-PCR reactions, 8 μg total RNA was reverse transcribed using iScript cDNA systhesis kit (Bio-Rad, 170-8891). qRT-PCR was performed using PowerUp SYBR Green Master Mix (Thermo Fisher Scientific, A25742) and GAPDH as an internal control. Each PCR was run in duplicate. Primer sequences were as follows: *Shh* forward 5′-AAA GCT GAC CCC TTT AGC CTA-3′, *Shh* reverse 5′-TTC GGA GTT TCT TGT GAT CTT CC-3′, *Ihh* forward 5′-CTC TTG CCT ACA AGC AGT TCA-3′, *Ihh* reverse 5′-CCG TGT TCT CCT CGT CCT T-3′, *Dhh* forward 5′-CTT GGC ACT CTT GGC ACT ATC-3′, *Dhh* reverse 5′-GAC CCC CTT GTT ACC CTC C-3′, *Gapdh* forward 5′-CCA AGG TGT CCG TCG TGG ATC T-3′, and *Gapdh* reverse 5′-GTT GAA GTC GCA GGA GAC AAC C-3′.

### Human tissue array of normal prostate and cancer

Images of human prostate tissues and cancers are obtained from the Human Protein Atlas (http://v15.proteinatlas.org/). Specific images for genes examined in this study can be found via the following links: CNN1 (calponin), normal prostate: http://www.proteinatlas.org/ENSG00000130176-CNN1/tissue/prostate, prostate cancer: http://www.proteinatlas.org/ENSG00000130176-CNN1/cancer/tissue/prostate+cancer; ACTA2 (actin, alpha 2, smooth muscle, aorta), normal prostate: http://www.proteinatlas.org/ENSG00000107796-ACTA2/tissue/prostate, prostate cancer: http://www.proteinatlas.org/ENSG00000107796-ACTA2/cancer/tissue/prostate+cancer; VIM (vimentin), normal prostate: http://www.proteinatlas.org/ENSG00000026025-VIM/tissue/prostate, prostate cancer: http://www.proteinatlas.org/ENSG00000026025-VIM/cancer/tissue/prostate+cancer.
